# Repeated Prostate Cancer Screening Using Prostate-Specific Antigen Testing and Magnetic Resonance Imaging: A Secondary Analysis of the STHLM3-MRI Randomized Clinical Trial

**DOI:** 10.1001/jamanetworkopen.2023.54577

**Published:** 2024-02-07

**Authors:** Tobias Nordström, Magnus Annerstedt, Axel Glaessgen, Stefan Carlsson, Mark Clements, Ahmad Abbadi, Henrik Grönberg, Fredrik Jäderling, Martin Eklund, Andrea Discacciati

**Affiliations:** 1 Department of Medical Epidemiology and Biostatistics, Karolinska Institutet, Stockholm, Sweden; 2 Department of Clinical Sciences, Danderyd Hospital, Karolinska Institutet, Stockholm, Sweden; 3 C-Medical Urology Odenplan, Stockholm, Sweden; 4 Department of Clinical Pathology and Cytology, Unilabs, Stockholm, Sweden; 5 Department of Urology, Karolinska University Hospital Solna, Stockholm, Sweden; 6 Department of Molecular Medicine and Surgery, Karolinska Institutet, Stockholm, Sweden; 7 Department of Diagnostic Radiology, Karolinska University Hospital, Stockholm, Sweden

## Abstract

**Importance:**

Magnetic resonance imaging (MRI) has been proposed to enhance the benefit-to-harm ratio of prostate cancer screening, but data on repeated screening outcomes are lacking.

**Objective:**

To describe outcomes of prostate-specific antigen (PSA)–based screening with MRI and prostate biopsies at repeat screening.

**Design, Setting, and Participants:**

This secondary analysis examined the population-based, screen-by-invitation STHLM3-MRI randomized clinical trial, which recruited Swedish men aged 50 to 74 years. Men were eligible for repeat screening at 2 to 3 years if they had PSA levels of 1.5 ng/mL or greater at trial inclusion, were randomized to the MRI-targeted group (including screening using biomarkers and MRI), and were not diagnosed with prostate cancer after the first screening round. Repeat screening was performed between November 10, 2021, and February 20, 2023. Data analysis was performed between May and August 2023.

**Intervention:**

Participants underwent blood sampling, including PSA testing. A biparametric MRI scan was performed if PSA levels were 3 ng/mL or greater, and men with lesions with a Prostate Imaging–Reporting and Data System (PI-RADS) score of 3 or greater were referred for targeted and systematic biopsies.

**Main Outcomes and Measures:**

The primary outcome was clinically significant prostate cancer (Gleason score of ≥3 + 4). Secondary outcomes included the proportion of men with clinically insignificant cancer (Gleason score of 6), the number of elevated PSA tests, MRI scans, and biopsy procedures.

**Results:**

Of 7609 men from the first screening round, 2078 (27.3%) were eligible for and were invited for rescreening. Among the invitees, 1500 (72.2%) participated. Their median age was 67 (IQR, 61-72) years. Of 1094 men with PSA levels between 1.5 and 2.9 ng/mL in the first screening round, 326 (29.8%) had levels of 3 ng/mL or greater in the second round. Overall, 667 men (44.5%) had PSA levels of 3 ng/mL or greater: 617 underwent MRI (92.5%), revealing 51 (7.6%) with equivocal lesions (PI-RADS score of 3) and 33 (4.9%) with suspicious lesions (PI-RADS score of ≥4). Only 10 of 383 men (2.6%) with a prior negative MRI result had a lesion with a PI-RADS score of 4 or greater. Among the 1500 rescreened men, 48 (3.2%) had a Gleason score of 3 + 4 or greater, including 19 (1.3%) with a score of 4 + 3 or greater and 11 (0.7%) with a score of 6.

**Conclusions and Relevance:**

In this secondary analysis of the STHLM3-MRI randomized clinical trial, cancer detection during the second screening round in biennial PSA and MRI-based prostate cancer screening was limited, and the detection of low-grade tumors remained low. A substantial proportion of men exhibited elevated PSA levels during rescreening, and a considerable portion of MRI scans performed lacked lesions suggestive of cancer. Future studies should explore strategies to reduce MRI-related resource use.

**Trial Registration:**

ClinicalTrials.gov Identifier: NCT03377881

## Key Points

**Question:**

What are the outcomes associated with repeated screening for prostate cancer using prostate-specific antigen testing and magnetic resonance imaging (MRI)?

**Findings:**

In this secondary analysis of the STHLM3-MRI randomized clinical trial of 1500 men who had a repeat test 2 to 3 years after their original prostate cancer screening, cancer with a Gleason score of 3 + 4 or greater was detected in 3% of men and a tumor with a Gleason score of 6 was detected in less than 1%. A high proportion of the performed MRI scans lacked lesions that were suspicious for cancer.

**Meaning:**

In repeated prostate cancer screening, detection of clinically significant cancer was limited and detection of low-grade cancer remained low; these findings suggest that strategies to reduce MRI-related resource use are needed.

## Teaser

This secondary analysis of a randomized clinical trial explores outcomes of prostate-specific antigen (PSA)–based screening with magnetic resonance imaging (MRI) and prostate biopsy at repeat screening 2 to 3 years later.

## Introduction

Prostate-specific antigen (PSA)–based screening for prostate cancer is associated with a decrease in prostate cancer–specific mortality.^1^ Potential overdiagnosis associated with PSA testing and traditional biopsies can be mitigated by an initial risk assessment (eg, using PSA), followed by magnetic resonance imaging (MRI) and then targeted and traditional biopsies. Level 1 evidence indicates that detection of low-grade cancers can be halved by avoiding biopsies in men with a PSA-positive result with negative MRI scans while maintaining sensitivity to detect clinically significant cancer.^2,3^ Consequently, clinical practice has changed rapidly, and many men now undergo MRI before biopsy.^4,5^

After a decade of consensus that population-based prostate cancer screening using PSA testing and standard biopsies is associated with high levels of overdiagnosis, projects for organizing screening with MRI have been initiated in Europe.^6,7^ The current evidence does not support the effectiveness of a single PSA test.^8^ Thus, guidelines and programs for prostate cancer early detection include repeat testing at various intervals, often conducted every 1 to 4 years.^4,5^ Nonetheless, the only available data are from the Göteborg 2 trial, which reported on a small subset of men who had undergone previous MRI, to support decision-making for repeat testing using MRI.^9^

Due to the detection of prevalent cancers in a first screening round, cancer detection in repeat rounds is expected to be lower. This expectation is supported by historical data using standard biopsies, in which only 1% of men invited to the second screening round in the Rotterdam section of the European Randomized Study of Screening for Prostate Cancer had a Gleason score of 7 or greater compared with 2% in the first round.^10^ To our knowledge, there are no data on outcomes of repeat prostate cancer screenings using strategies including MRI. Here, we describe findings for men who received a second screening 2 to 3 years after the first screening, using a contemporary approach with PSA testing, MRI, and combined targeted and standard prostate biopsies.

## Methods

### STHLM3-MRI Study Design

This study is a secondary analysis of STHLM3-MRI, a prospective and population-based clinical trial that used a combined paired screen-positive and randomized design to compare multiple diagnostic strategies within the same trial.^11^ The trial was approved by the Swedish Ethical Review Authority and registered with ClinicalTrials.gov (NCT03377881). The trial protocol and statistical analysis plan are available in Supplement 1. All participants provided written informed consent. The study followed the Consolidated Standards of Reporting Trials (CONSORT) reporting guideline.

Men aged 50 to 74 years living in Stockholm County, Sweden, were randomly selected by Statistics Sweden and invited by mail to participate in the study. Men with a previous prostate cancer diagnosis, a prostate biopsy within 60 days before invitation, any contraindications for MRI, or severe illness (ie, metastatic cancer, severe cardiovascular disease, or dementia) were deemed to not be eligible for prostate cancer screening and participation.^11^ Participants were asked to give blood for PSA testing and Stockholm3 blood testing. Participants were randomly assigned (2:3) to receive either traditional screening with systematic prostate biopsy (standard group) or to an MRI-based strategy with biparametric MRI, followed by MRI-targeted and systematic biopsy for men with a positive MRI result (experimental group).

In the STHLM3-MRI trial, men with PSA levels less than 1.5 ng/mL were considered to have a very low risk of clinically significant cancer and were recommended to return for a second screening at 6 years.^11^ If PSA levels were 1.5 ng/mL or greater, the Stockholm3 test was performed. Men with elevated PSA levels (≥3 ng/mL) or an elevated Stockholm3 risk score (≥0.11) were considered to have elevated risk and were recommended to undergo workup as defined by their study group, including clinical follow-up in the case of no cancer detected in men with a positive MRI result. The remaining men (with PSA levels ≥1.5 but <3 ng/mL and a Stockholm3 risk score <0.11) were considered to not have elevated risk of prostate cancer and were recommended to have repeat testing after 2 years. Randomization occurred at different stages: men with elevated risk on the first screening round blood test (elevated PSA levels or Stockholm3 risk score) were randomized immediately after blood analysis and referred for study interventions as indicated by the study group; the remaining men (without elevated PSA levels and without elevated Stockholm3 risk score) were randomized to the standard or experimental group before they were invited to the second screening round.

### Participant Rescreening

Invitations for a second screening round were sent 2 to 3 years after original study participation to men randomized to the experimental group who had PSA levels of 1.5 ng/mL or greater at the first screening round and did not have a prostate cancer diagnosis at the time of rescreening. The first rescreening for men with PSA levels less than 1.5 ng/mL is planned for 6 years after initial testing; thus, these men were not invited for the second screening round. Men were not reinvited if they had died since the first screening round, had a prostate cancer diagnosis, had emigrated, had protected identity, or did not comply with the initial screening round interventions such as MRI or biopsy procedures.

Study participants reconsented to a second round of screening through a secure web portal, where eligibility was assessed and a digital referral for blood sampling was created. Repeat screening was performed between November 10, 2021, and February 20, 2023.

Study interventions followed the protocol of the first screening round.^11^ Men with an elevated blood test result (PSA levels ≥3 ng/mL or a Stockholm3 risk score ≥0.11) were referred for MRI and biopsy procedures. To investigate outcomes after repeated testing when using PSA testing and an MRI-targeted approach, Stockholm3 test results and any interventions indicated due to an elevated Stockholm3 risk score were ignored.

### Blood Analysis

Twelve milliliters of EDTA blood plasma was drawn from each participant at any of 60 local laboratories in the Stockholm region and analyzed for PSA levels and Stockholm3 risk score at A3P Biomedical in Uppsala, Sweden. Prostate-specific antigen was analyzed using the same technique as in the first screening round (Kryptor Compact Plus; Thermo Fisher Scientific).

### MRI and Biopsy Procedures

Men with elevated risk of prostate cancer as deemed by blood analyses were referred for biparametric MRI using a 1.5T Magnetom (Siemens Healthcare) or 3T Signa (GE Healthcare) scanner without an endorectal coil. T2-weighted and diffusion-weighted images were acquired by use of a short (<10-minute) protocol. Areas suggestive of prostate cancer were graded according to a modification of the Prostate Imaging–Reporting and Data System (PI-RADS), version 2.1.^11^ The MRI protocol and reading procedures were essentially identical between the first and second screening rounds.

Transrectal or transperineal MRI-ultrasound fusion equipment (BK Fusion; BK Medical) was used to sample 3 to 4 biopsy cores from each suspicious lesion. A 10- to 12-core systematic biopsy was also performed in patients undergoing MRI-targeted biopsy in the same session by the same urologist. The Gleason score, International Society of Urological Pathology (ISUP) grade, percentage with Gleason grade 4 in cancerous tissue, and size (in millimeters) of cancer findings were reported for each biopsy according to ISUP guidelines.^12^

### Outcomes

The primary outcome was the proportion of men participating in the second screening round with clinically significant prostate cancer detected, defined as a Gleason score of 3 + 4 or greater (ISUP grade ≥2). Secondary outcomes included the proportion of men with clinically insignificant cancer, defined as a Gleason score of 3 + 3 (ISUP grade 1), the number of elevated PSA tests, MRI scans, and biopsy procedures.

### Statistical Analysis

Study participant characteristics are summarized using medians (IQRs) for continuous variables and proportions for categorical variables. Two-sided 95% CIs for proportions were calculated using the binomial exact method. We calculated the proportion of invited men who participated, had PSA results greater than 3 ng/mL, and who, following an elevated PSA test, underwent MRI and biopsy procedures, had a benign biopsy, or were diagnosed with insignificant, clinically significant, or high-grade (Gleason score of ≥4 + 3) prostate cancer. We normalized those proportions and their corresponding 95% CIs to a population of 10 000 invited men. Given the descriptive nature of this study, 95% CIs were not adjusted for multiplicity, and caution should be applied when evaluating their joint statistical level. All analyses were done in R, version 4.3.1 (R Project for Statistical Computing). Data analysis was performed between May and August 2023.

## Results

Of the 7609 men randomized to the experimental group at the first screening, 2078 (27.3%) were eligible for invitation to a second screening round (Figure). Of these eligible invitees, 1500 (72.2%) accepted and underwent blood testing. Their median age was 67 (IQR, 61-72) years (Table 1). Nonparticipants and participants shared similar characteristics in terms of age, total PSA values in the first screening round, prostate biopsy history, and family history of prostate cancer. There were no substantial differences in participation rate by prostate cancer risk in the first screening round (eTable 1 in Supplement 2).

**Figure.**
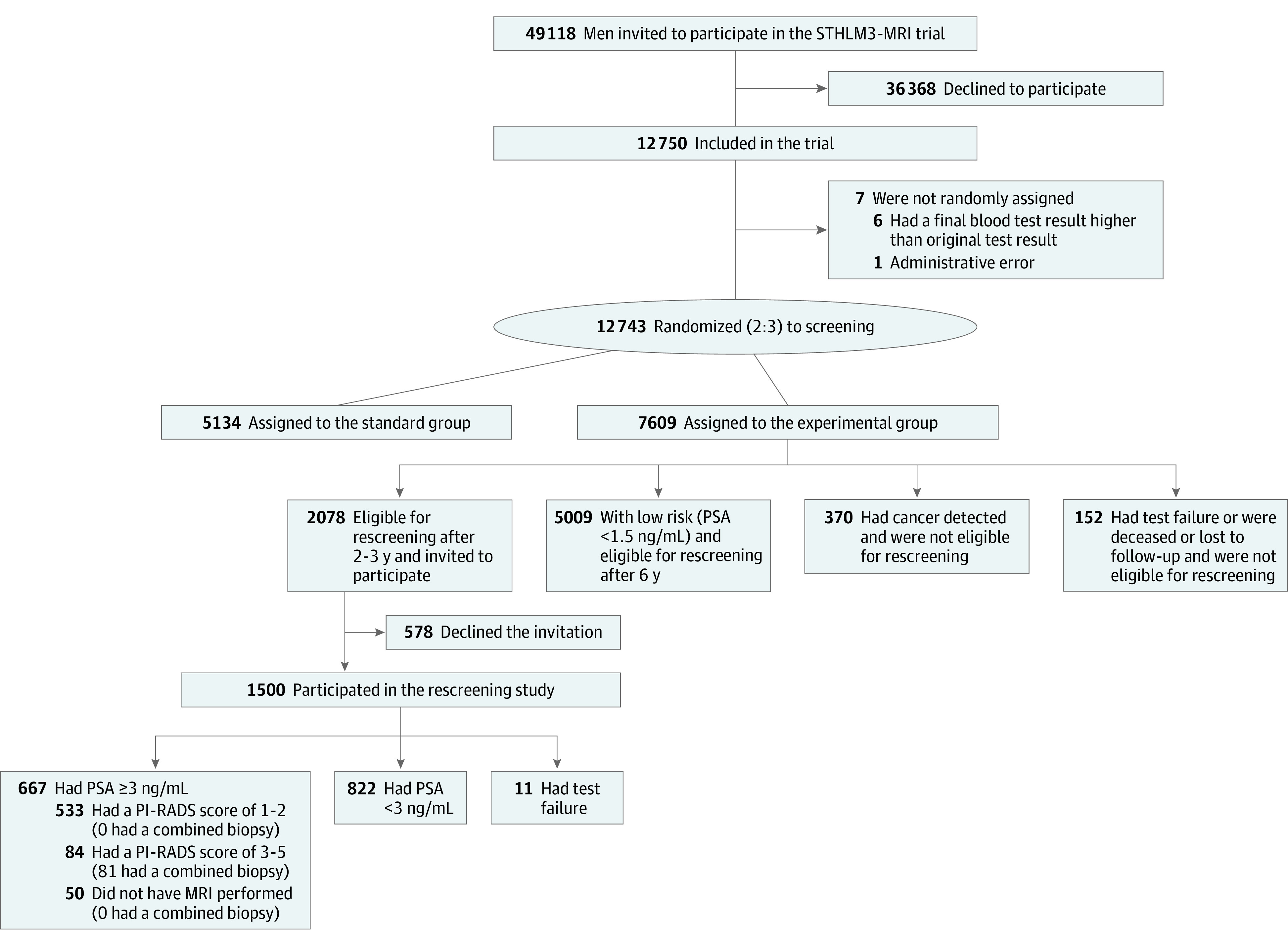
Profile of the Population-Based, Screen-by-Invitation STHLM3-MRI Randomized Clinical Trial MRI indicates magnetic resonance imaging; PI-RADS, Prostate Imaging–Reporting and Data System; and PSA, prostate-specific antigen.

**Table 1.** Participant Characteristics^a^

Characteristic	Participants in second screening round (N = 1500)	PSA level, ng/mL		Test failures (n = 11)
		<3 (n = 822)	≥3 (n = 667)	
Age, median (IQR), y	67 (61-72)	66 (60-71)	68 (63-72)	68 (64-71)
PSA level at inclusion				
Median (IQR), ng/mL	2.8 (2.1-4.0)	2.2 (1.7-2.5)	4.2 (3.5-5.6)	NA
Missing, No.	11	0	0	11
Previous biopsy procedure				
Yes	230 (16.3)	61 (7.9)	168 (26.7)	1 (10.0)
No	1179 (83.7)	708 (92.1)	462 (73.3)	9 (90.0)
Missing, No.	91	53	37	1
Family history of prostate cancer				
Yes	269 (20.2)	156 (21.2)	111 (19.0)	2 (18.2)
No	1063 (79.8)	581 (78.8)	473 (81.0)	9 (81.8)
Missing, No.	168	85	83	0
Invitation group^b^				
Not elevated risk at first screening	850 (56.7)	599 (72.9)	243 (36.4)	8 (72.7)
Elevated risk at first screening and negative MRI result	587 (39.1)	201 (24.5)	383 (57.4)	3 (27.3)
Elevated risk at first screening and negative biopsy result	63 (4.2)	22 (2.7)	41 (6.1)	0
Time difference between first and second screening rounds, median (IQR), y	2.4 (2.4-2.7)	2.4 (2.4-2.6)	2.4 (2.4-2.7)	2.6 (2.5-2.8)

Abbreviations: MRI, magnetic resonance imaging; NA, not applicable; PSA, prostate-specific antigen.

^a^ Unless indicated otherwise, values are the No. (%) of participants.

^b^ Elevated risk was defined as PSA levels of 3 ng/mL or greater or a Stockholm3 risk score of 0.11 or greater.

### Blood-Test Results

The median PSA level among rescreened men was 2.8 (IQR, 2.1-4.0) ng/mL (eFigures 1 and 2 in Supplement 2). Of the 1500 participants, 667 (44.5% [95% CI, 41.9%-47.0%]) had PSA levels of 3 ng/mL or greater at rescreening.

Of 1094 men with PSA levels of 1.5 to 2.9 ng/mL in the first screening round, 326 (29.8% [95% CI, 27.1%-32.6%]) had PSA levels of 3 ng/mL or greater at rescreening and 119 men (10.9% [95% CI, 9.1%-12.9%]) were reclassified to PSA levels less than 1.5 ng/mL. Conversely, of 406 men with PSA levels of 3 ng/mL or greater at the first screening and no cancer detected, 341 (84.0%) had PSA levels of 3 ng/mL or greater at rescreening (Table 2). Of the 1500 participants, 390 (26.0%) changed risk classification groups (PSA levels <3 vs ≥3 ng/mL).

**Table 2.** Blood-Test Reclassification From First to Second Screening After 2 to 3 Years^a^

	PSA level at second screening, ng/mL			Test failures	Total
	<1.5	1.5-2.9	≥3		
PSA level at first screening, ng/mL					
1.5-2.9	119 (10.9)	639 (58.4)	326 (29.8)	10 (0.9)	1094 (100)
≥3	9 (2.2)	55 (13.5)	341 (84.0)	1 (0.2)	406 (100)
Total	128 (8.5)	694 (46.3)	667 (44.5)	11 (0.7)	1500 (100)

Abbreviation: PSA, prostate-specific antigen.

^a^ Values are presented as No. (%) of participants. Men with PSA levels less than 1.5 ng/mL at the first screening were not invited to rescreening in this study but are planned for rescreening after 6 years.

### MRI Results

Of 667 men with PSA levels of 3 ng/mL or greater at the second screening, 617 (92.5%) underwent MRI. Adherence to an MRI recommendation was similar regardless of the risk group after the first screening round. A total of 533 men (79.9% [95% CI, 76.7%-82.9%]) had no findings that were suspicious for prostate cancer, 51 (7.6% [95% CI, 5.7%-9.9%]) had equivocal MRI findings (PI-RADS score of 3), and 33 (4.9% [95% CI, 3.4%-6.9%]) had at least 1 suspicious lesion classified as a PI-RADS score of 4 or 5. Only 10 of 667 men (1.5% [95% CI, 0.7%-2.7%]) had a lesion with a PI-RADS score of 5 at the second screening round, and all of these lesions were found in men who did not undergo MRI in the first screening round (Table 3).

**Table 3.** Findings in 1500 Men With PSA Levels of 1.5 ng/mL or Greater at the First Screening and Referred for MRI 2 to 3 Years After the Initial Screening^a^

	PI-RADS score				MRI not performed	Total
	≤2	3	4	5		
Invitation group^b^						
Not elevated risk at first screening	177 (72.8)	28 (11.5)	13 (5.3)	10 (4.1)	15 (6.2)	243 (100)
Elevated risk at first screening, negative MRI result	321 (83.8)	21 (5.5)	10 (2.6)	0	31 (8.1)	383 (100)
Elevated risk at first screening, negative biopsy result	35 (85.4)	2 (4.9)	0	0	4 (9.8)	41 (100)
Total	533 (79.9)	51 (7.6)	23 (3.4)	10 (1.5)	50 (7.5)	667 (100)

Abbreviations: MRI, magnetic resonance imaging; PI-RADS, Prostate Imaging–Reporting and Data System; PSA, prostate-specific antigen.

^a^ Values are presented as No. (%) of participants. If PSA at rescreening exceeded 3 ng/mL, MRI was indicated.

^b^ Elevated risk was defined as PSA levels of 3 ng/mL or greater or a Stockholm3 risk score of 0.11 or greater.

The proportion of MRI scans with lesions suspicious for cancer (PI-RADS score ≥4) was low among men with a previous negative MRI result (10 of 383 [2.6%]). No lesions with PI-RADS scores of 4 or 5 were found among men with a previous positive MRI result followed by benign findings at biopsy.

The detection rate of Gleason scores of 7 or greater was 45.8% (22 of 48 [95% CI, 31.4%-60.8%]) in men with a PI-RADS score of 3, 69.6% (16 of 23 [95% CI, 47.1%-86.8%]) in men with a PI-RADS score of 4, and 100% (10 of 10) in men with lesions with a PI-RADS score of 5. The detection rates of tumors with a Gleason score of 6 were similar in men with lesions with a PI-RADS score of 3 or 4 (8 of 48 [16.7%] and 3 of 23 [13.0%]; eTable 2 in Supplement 2).

### Cancer Detection

Of the 84 men with lesions with a PI-RADS score of 3 or greater, 81 (96.4%) underwent a biopsy procedure. Of the 1500 rescreened men, 48 (3.2% [95% CI, 2.4%-4.2%]) had PSA levels of 3 ng/mL or greater and prostate cancer was detected (Gleason score of ≥7), corresponding to 48 of the 81 biopsied men (59.3%). A Gleason score of 6 was detected in 0.7% (11 of 1500 [95% CI, 0.4%-1.3%]) of rescreened men and a score of 4 + 3 or greater was detected in 1.3% (19 of 1500 [95% CI, 0.8%-2.0%]) (Table 4). Of the clinically significant cancers, 38 of 48 (79.2%) were found in men with PSA levels of 3 to 4.9 ng/mL (eTable 3 in Supplement 2).

**Table 4.** Findings in 1500 Men With PSA Levels of 1.5 ng/mL or Greater at the First Screening and Referred for Prostate Biopsy 2 to 3 Years After the Initial Screening^a^

	ISUP grade group						Biopsy not performed	Total
	Benign	1	2	3	4	5		
Invitation group^b^								
Not elevated risk at first screening (n = 850)	13 (25.5)	6 (11.8)	20 (39.2)	5 (9.8)	1 (2.0)	6 (11.8)	0	51 (100)
Elevated risk at first screening, negative MRI result (n = 587)	8 (25.8)	5 (16.1)	9 (29.0)	1 (3.2)	0	5 (16.1)	3 (9.7)	31 (100)
Elevated risk at first screening, negative biopsy result (n = 63)	1 (50.0)	0	0	1 (50.0)	0	0	0	2 (100)
Total	22 (26.2)	11 (13.1)	29 (34.5)	7 (8.3)	1 (1.2)	11 (13.1)	3 (3.6)	84 (100)

Abbreviations: ISUP, International Society of Urological Pathology; MRI, magnetic resonance imaging; PI-RADS, Prostate Imaging–Reporting and Data System; PSA, prostate-specific antigen.

^a^ Values are presented as No. (%) of participants. Participants with any PI-RADS MRI finding of 3 or greater underwent a combined biopsy procedure including 3 to 4 targeted biopsies per lesion with the addition of 10 to 12 systematic biopsies to the dorsal prostate.

^b^ Elevated risk was defined as PSA levels of 3 ng/mL or greater or a Stockholm3 risk score of 0.11 or greater.

The number of participants in the experimental group (n = 7609) with a Gleason score of 7 or greater detected when using PSA testing and MRI as described earlier was higher in the first screening round^3^ than in the second (192 [2.5%] vs 48 [0.6%]; *P* < .001). However, it should be noted that participants with PSA levels less than 1.5 ng/mL in the first screening round were not invited to participate in the second round; retesting is planned for these men after 6 years from the first screening round, as indicated in the study protocol (Supplement 1).

### Resource Use

eFigure 3 in Supplement 2 illustrates the number of procedures performed and cancers detected in a second screening round using targeted plus systematic prostate biopsies in men with a positive MRI result and elevated PSA levels (≥3 ng/mL), normalized to a population of 10 000 invited men.

## Discussion

Early detection of prostate cancer using PSA testing and MRI has previously been shown to maintain detection of clinically significant disease and to decrease the high rates of overdiagnosis associated with traditional workup.^2,3,13^ However, because no benefit for prostate cancer–specific mortality has been observed from a single PSA test, the performance of prostate cancer detection in the repeat testing setting is crucial.^8^ In this study, compliance with repeat prostate cancer screening was reasonably high, and a substantial proportion of men who had repeat screening were reclassified as having elevated risk and thus offered workup. In line with this finding, the number of MRI scans in repeat screening was high, whereas the proportion of MRI scans that indicated suspicion of prostate cancer was low (84 of 617 [13.6%]). Nonetheless, clinically significant cancer was detected for 48 (3.2%) of the 1500 eligible men in the STHLM3-MRI trial who participated in the second screening round, although only a few of the detected clinically significant cancers were high grade (Gleason score ≥4 + 4; 12 [0.8%]). Men with low PSA levels (<1.5 ng/mL) in the first screening round will only be offered retesting in a third screening round (at 6 years after initial screening), so the long-term cancer detection rate in prostate cancer screening remains to be shown.

Nearly three-quarters of men (1500 [72.2%]) accepted the invitation to a second screening round. This participation rate is substantially higher than that for a first screening round in this and previous Swedish population-based prostate cancer detection studies.^11,14,15^ The high compliance is likely due to the eligibility criteria for the second screening round (men with PSA levels ≥1.5 ng/mL participating and complying in the first round). Nonetheless, we believe that this high participation rate is consistent with an understanding in the general population of the importance of repeat screening and that potential efforts to increase compliance may be primarily focused to initial nonresponders in a screening program.

We found that 390 of the 1500 participants (26.0%) changed risk classification groups at repeat screening after 2 to 3 years. It is unknown to what extent this represents true risk progression or if it primarily reflects the variable nature of PSA measurements. As a consequence of the eligibility criteria in the second screening round, in which men with originally low PSA levels (<1.5 ng/mL) were not invited to participate (Figure), the number of men with elevated PSA levels was high, meaning that a high proportion of men underwent additional interventions such as MRI. In this study, 667 participants (44.5%) had a PSA-based indication for MRI (≥3 ng/mL).

Of the 617 MRI scans performed in the second round, 533 (86.4%) lacked any suspicious findings. For the 352 men who underwent MRI in the first screening round, the corresponding value was 321 (91.2%). The high proportion of negative MRI results is striking, and it would lead to an overutilization of MRI resources if used for biannual prostate cancer screening in combination with PSA levels of 3 ng/mL or greater as the cutoff for a single-biomarker strategy for further workup. This supports the use of reflex testing in men with moderately elevated PSA levels in a screening program.

The true long-term prostate cancer detection rate remains to be shown while men with PSA levels of less than 1.5 ng/mL will only be invited for repeat testing in subsequent screening rounds. Nonetheless, we found that 3.2% of men who came for rescreening after 2 to 3 years had clinically significant cancer detected when using a contemporary MRI-based detection strategy, representing a nonnegligible detection rate. However, the number of participants in the STHLM3-MRI experimental group with clinically significant cancer detected after PSA testing and MRI was substantially higher in the first screening round (192 of 7609 [2.5%])^3^ than in the second (48 of 7609 [0.6%]). Furthermore, only 19 of 48 (39.6%) clinically significant cancers detected at rescreening had a Gleason score of 4 + 3 or greater, illustrating a limited detection rate when using a more stringent definition for clinically significant cancer.^16^

It is reassuring that the detection rate of low-grade tumors remained low at rescreening. This finding indicates that avoiding biopsy in men with negative MRI results reduced overdetection at rescreening, as shown in the first screening round.

Our findings are in line with those from the Göteborg 2 trial in men with a previous MRI scan. The Göteborg 2 investigators reported that 89% of MRI scans performed in men with a previous MRI scan remained negative, with the corresponding proportion in our study being 91.2%. Since the Göteborg 2 trial has not reported rescreening data including men with PSA levels of 3 ng/mL or less at the first screening, further comparisons between that study and ours remain challenging. However, our data corroborate the suggestion that a 2-year interval for rescreening seems safe in light of the limited cancer detection at repeat screening.^9^

### Strengths and Limitations

Our study is the first to report a comprehensive summary of findings in prostate cancer rescreening using contemporary detection strategies including MRI. Key strengths of our trial include the large number of participants invited from a population-based sample of screening participants, the use of a short biparametric MRI protocol suitable for screening purposes, and well-defined interventions. However, our study is not devoid of limitations. First, these data only represent findings from men with PSA levels of 1.5 ng/mL or greater at the first screening round invited to a second one after 2 to 3 years, and they lack long-term outcomes such as prostate cancer mortality. Consequently, any extrapolation to subsequent screening rounds and long-term results must be made with caution. We will invite men with low PSA levels (<1.5 ng/mL) to rescreen 6 years after original study inclusion. Second, cancer detection in men with a negative MRI result at rescreening is unknown. However, a high negative predictive value of negative MRI has been reported.^17^ We argue that the safety of omitting prostate biopsies in men with a negative MRI result is strengthened by the low proportion of men transitioning from an originally negative MRI result to positive MRI findings at rescreening in this trial. Third, 55 men with PSA levels of 3 ng/mL or less in the experimental group had clinically significant prostate cancer detected during the first testing round in our study.^11^ If we assumed that some of those men had PSA levels of 3 ng/mL or greater in the second screening round, then this would affect the detection rate. In particular, although there were few, detection of those cancers later would increase the detection rate reported here. Fourth, although men invited to participate in STHLM3-MRI represent a population-based sample, self-selection mechanisms might limit the external validity of the results presented here.

## Conclusions

In this secondary analysis of the STHLM3-MRI randomized clinical trial, we found that participation in a first prostate cancer rescreening after 2 to 3 years was high. The detection of clinically significant cancer was limited, but longer-term detection rates remain to be shown in future screening rounds, including among men with lower PSA levels. The proportion of MRI results without pathologic lesions was observed to be very high. Therefore, future studies should outline alternatives for improved risk stratification, including personalized screening intervals and interventions such as the use of reflex testing.

## Notes

Supplement 1.
Trial Protocol and Statistical Analysis Plan

Supplement 2. **eTable 1.** Characteristics of Invited, Participating, and Nonparticipating Men in a Second Screening Round **eTable 2.** Findings in 1500 Men With Prostate-Specific Antigen Levels ≥1.5 ng/mL at First Screening and Referred to Prostate Biopsy 2 to 3 Years After the Initial Screening, Stratified by Magnetic Resonance Imaging Findings **eTable 3.** Findings in 1500 Men With Prostate-Specific Antigen (PSA) Levels ≥1.5 ng/mL at First Screening and Referred to Prostate Biopsy 2 to 3 Years After the Initial Screening, Stratified by PSA Level **eFigure 1.** Distribution of Prostate-Specific Antigen (PSA) Levels in 1500 Men Undergoing a Second Prostate Cancer Screening After 2 to 3 Years, Overall and Stratified by Invitation Group at Second Screening **eFigure 2.** Scatterplot of Prostate-Specific Antigen (PSA) Levels at Second Vs First Screening in 1500 Men Undergoing a Second Screening After 2 to 3 Years, Overall and Stratified by Invitation Group at Second Screening **eFigure 3.** Number of Participants, Elevated Prostate-Specific Antigen (PSA) Test Results, Magnetic Resonance Imaging (MRI) and Biopsy Procedures Performed, and Detected Cancers Normalized to a Population of 10 000 Men Invited to a Second Screening

Supplement 3.
Data Sharing Statement
