# Supplementary material for: Repeated Prostate Cancer Screening Using Prostate-Specific Antigen Testing and Magnetic Resonance Imaging: A Secondary Analysis of the STHLM3-MRI Randomized Clinical Trial
Source: JAMA Netw Open. 2024 Feb 7;7(2):e2354577. doi: 10.1001/jamanetworkopen.2023.54577 (PMC10851096; doi:10.1001/jamanetworkopen.2023.54577)
Supplement: Supplement 2. — eTable 1. Characteristics of Invited, Participating, and Nonparticipating Men in a Second Screening Round eTable 2. Findings in 1500 Men With Prostate-Specific Antigen Levels ≥1.5 ng/mL at First Screening and Referred to Prostate Biopsy 2 to 3 Years After the Initial Screening, Stratified by Magnetic Resonance Imaging Findings eTable 3. Findings in 1500 Men With Prostate-Specific Antigen (PSA) Levels ≥1.5 ng/mL at First Screening and Referred to Prostate Biopsy 2 to 3 Years After the Initial Screening, Stratified by PSA Level eFigure 1. Distribution of Prostate-Specific Antigen (PSA) Levels in 1500 Men Undergoing a Second Prostate Cancer Screening After 2 to 3 Years, Overall and Stratified by Invitation Group at Second Screening eFigure 2. Scatterplot of Prostate-Specific Antigen (PSA) Levels at Second Vs First Screening in 1500 Men Undergoing a Second Screening After 2 to 3 Years, Overall and Stratified by Invitation Group at Second Screening eFigure 3. Number of Participants, Elevated Prostate-Specific Antigen (PSA) Test Results, Magnetic Resonance Imaging (MRI) and Biopsy Procedures Performed, and Detected Cancers Normalized to a Population of 10 000 Men Invited to a Second Screening [file jamanetwopen-e2354577-s002.pdf]

Supplementary Online Content

Nordström T, Annerstedt M, Glaessgen A, et al. Repeated prostate cancer screening using prostate-specific antigen testing and magnetic resonance imaging: a secondary analysis of the STHLM3-MRI randomized clinical trial. *JAMA Netw Open*. 2024;7(2):e2354577. doi:10.1001/jamanetworkopen.2023.54577

eTable 1. Characteristics of Invited, Participating, and Nonparticipating Men in a Second Screening Round

eTable 2. Findings in 1500 Men With Prostate-Specific Antigen Levels ≥1.5 ng/mL at First Screening and Referred to Prostate Biopsy 2 to 3 Years After the Initial Screening, Stratified by Magnetic Resonance Imaging Findings

eTable 3. Findings in 1500 Men With Prostate-Specific Antigen (PSA) Levels ≥1.5 ng/mL at First Screening and Referred to Prostate Biopsy 2 to 3 Years After the Initial Screening, Stratified by PSA Level

eFigure 1. Distribution of Prostate-Specific Antigen (PSA) Levels in 1500 Men Undergoing a Second Prostate Cancer Screening After 2 to 3 Years, Overall and Stratified by Invitation Group at Second Screening

eFigure 2. Scatterplot of Prostate-Specific Antigen (PSA) Levels at Second Vs First Screening in 1500 Men Undergoing a Second Screening After 2 to 3 Years, Overall and Stratified by Invitation Group at Second Screening

eFigure 3. Number of Participants, Elevated Prostate-Specific Antigen (PSA) Test Results, Magnetic Resonance Imaging (MRI) and Biopsy Procedures Performed, and Detected Cancers Normalized to a Population of 10 000 Men Invited to a Second Screening

This supplementary material has been provided by the authors to give readers additional information about their work

© 2024 Nordström T et al. *JAMA Network Open.*

eTable 1. Characteristics of Invited, Participating, and Nonparticipating Men in a Second Screening Round

	**Invited to second screening round**, n = 2078[1]	**Participants**, n = 1,500[1]	**Non-participants**, n = 578[1]
Age (years)[2]	64 (58, 69)	64 (59, 69)	64 (57, 68)
PSA at inclusion (ng/ml)[2]	2.26 (1.80, 3.15)	2.27 (1.79, 3.14)	2.24 (1.80, 3.20)
Stockholm3 at inclusion[2]	8.0 (5.0, 11.0)	8.0 (5.0, 11.0)	8.0 (5.0, 11.0)
Previous biopsy procedure[2]			
Yes	191 (9.5%)	139 (9.6%)	52 (9.4%)
No	1,811 (90%)	1,310 (90%)	501 (91%)
Missing	76	51	25
Family history of prostate cancer[2]			
Yes	312 (17%)	232 (18%)	80 (16%)
No	1,500 (83%)	1,086 (82%)	414 (84%)
Missing	266	182	84
5-a-reductase inhibitor[2]			
Yes	69 (3.4%)	51 (3.4%)	18 (3.2%)
No	1,990 (97%)	1,440 (97%)	550 (97%)
Missing	19	9	10
Invitation group[3]			
Not elevated risk at first screening	1,193 (57%)	850 (57%)	343 (59%)
Elevated risk at first screening, MRI-negative	782 (38%)	587 (39%)	195 (34%)
Elevated risk at first screening, negative biopsy	103 (5.0%)	63 (4.2%)	40 (6.9%)

[1]Median (IQR); n (%)

[2]At first screening round.

[3]At second screening round.

© 2024 Nordström T et al. *JAMA Network Open.*

eTable 2. Findings in 1500 Men With Prostate-Specific Antigen Levels ≥1.5 ng/mL at First Screening and Referred to Prostate Biopsy 2 to 3 Years After the Initial Screening, Stratified by Magnetic Resonance Imaging Findings

	ISUP from Combined Biopsy (SBx and/or TBx), No (%)							
	Benign	1	2	3	4	5	Biopsy not performed	**Total**
**PI-RADS score**								
3	18 (35.3%)	8 (15.7%)	15 (29.4%)	1 (2.0%)	1 (2.0%)	5 (9.8%)	3 (5.9%)	51 (100.0%)
4	4 (17.4%)	3 (13.0%)	10 (43.5%)	3 (13.0%)	0 (0.0%)	3 (13.0%)	0 (0.0%)	23 (100.0%)
5	0 (0.0%)	0 (0.0%)	4 (40.0%)	3 (30.0%)	0 (0.0%)	3 (30.0%)	0 (0.0%)	10 (100.0%)
**Total**	22 (26.2%)	11 (13.1%)	29 (34.5%)	7 (8.3%)	1 (1.2%)	11 (13.1%)	3 (3.6%)	84 (100.0%)

Note: Participants with any PI-RADS≥3 MRI finding underwent a combined biopsy procedure including 3-4 targeted biopsies per lesion with the addition of 10-12 systematic biopsies to the dorsal prostate

© 2024 Nordström T et al. *JAMA Network Open.*

eTable 3. Findings in 1500 Men With Prostate-Specific Antigen (PSA) Levels ≥1.5 ng/mL at First Screening and Referred to Prostate Biopsy 2 to 3 Years After the Initial Screening, Stratified by PSA Level

	ISUP from Combined Biopsy (SBx and/or TBx), No (%)							
	Benign	1	2	3	4	5	Biopsy not performed	**Total**
**PSA at second screening**								
3-4.9 ng/ml (n=442)	16 (24.6%)	10 (15.4%)	25 (38.5%)	5 (7.7%)	1 (1.5%)	7 (10.8%)	1 (1.5%)	65 (100.0%)
5-9.9 ng/ml (n=208)	6 (33.3%)	1 (5.6%)	4 (22.2%)	2 (11.1%)	0 (0.0%)	4 (22.2%)	1 (5.6%)	18 (100.0%)
≥10 ng/ml (n=17)	0 (0.0%)	0 (0.0%)	0 (0.0%)	0 (0.0%)	0 (0.0%)	0 (0.0%)	1 (100.0%)	1 (100.0%)
**Total**	22 (26.2%)	11 (13.1%)	29 (34.5%)	7 (8.3%)	1 (1.2%)	11 (13.1%)	3 (3.6%)	84 (100.0%)

Note: Participants with any PI-RADS≥3 MRI finding underwent a combined biopsy procedure including 3-4 targeted biopsies per lesion with the addition of 10-12 systematic biopsies to the dorsal prostate

© 2024 Nordström T et al. *JAMA Network Open.*

eFigure 1. Distribution of Prostate-Specific Antigen (PSA) Levels in 1500 Men Undergoing a Second Prostate Cancer Screening After 2 to 3 Years, Overall and Stratified by Invitation Group at Second Screening

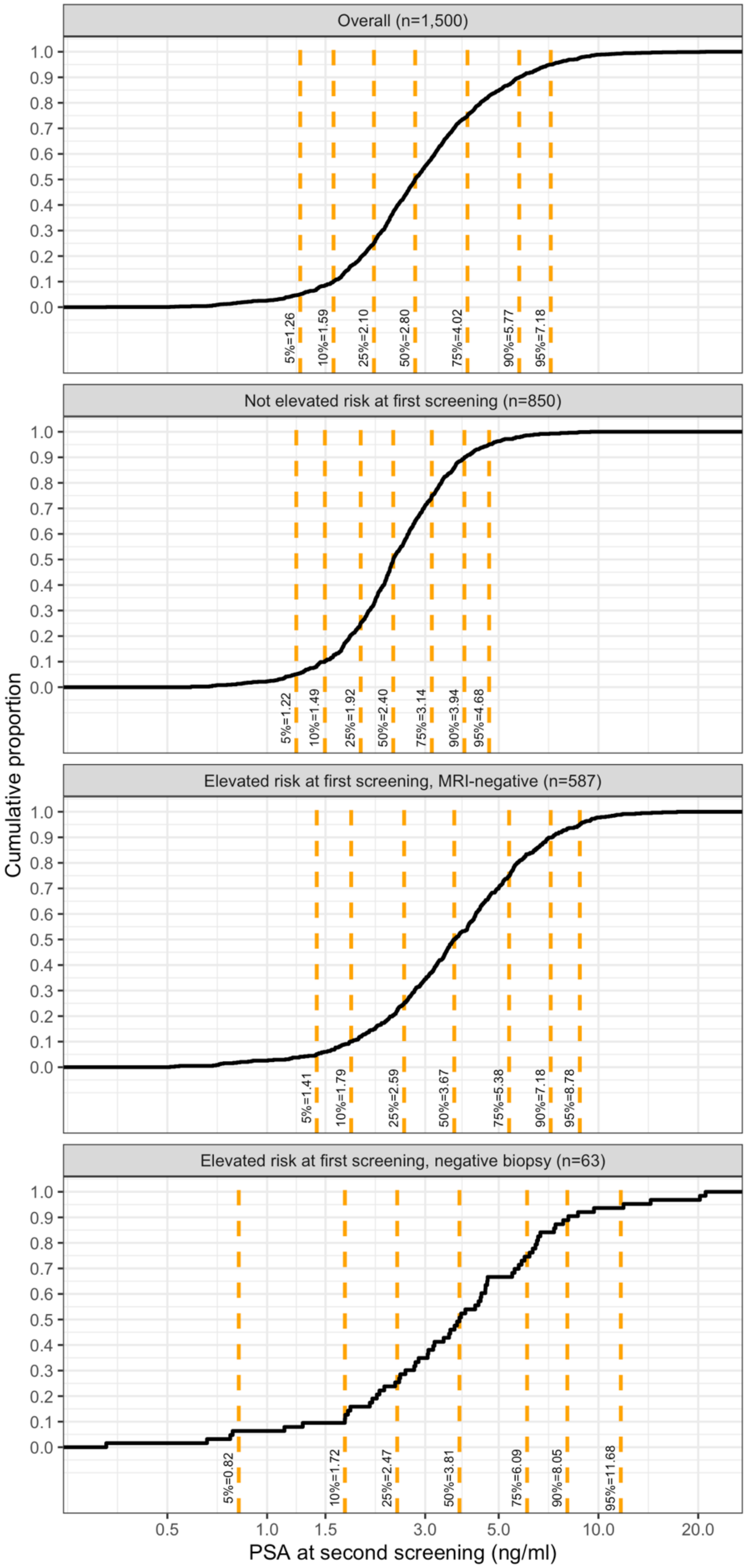

n=11 data points were excluded because of test failure.
Orange dashed lines indicate 5, 10, 25, 50, 75, 90 and 95th percentiles of the PSA distribution.

© 2024 Nordström T et al. *JAMA Network Open.*

eFigure 2. Scatterplot of Prostate-Specific Antigen (PSA) Levels at Second Vs First Screening in 1500 Men Undergoing a Second Screening After 2 to 3 Years, Overall and Stratified by Invitation Group at Second Screening

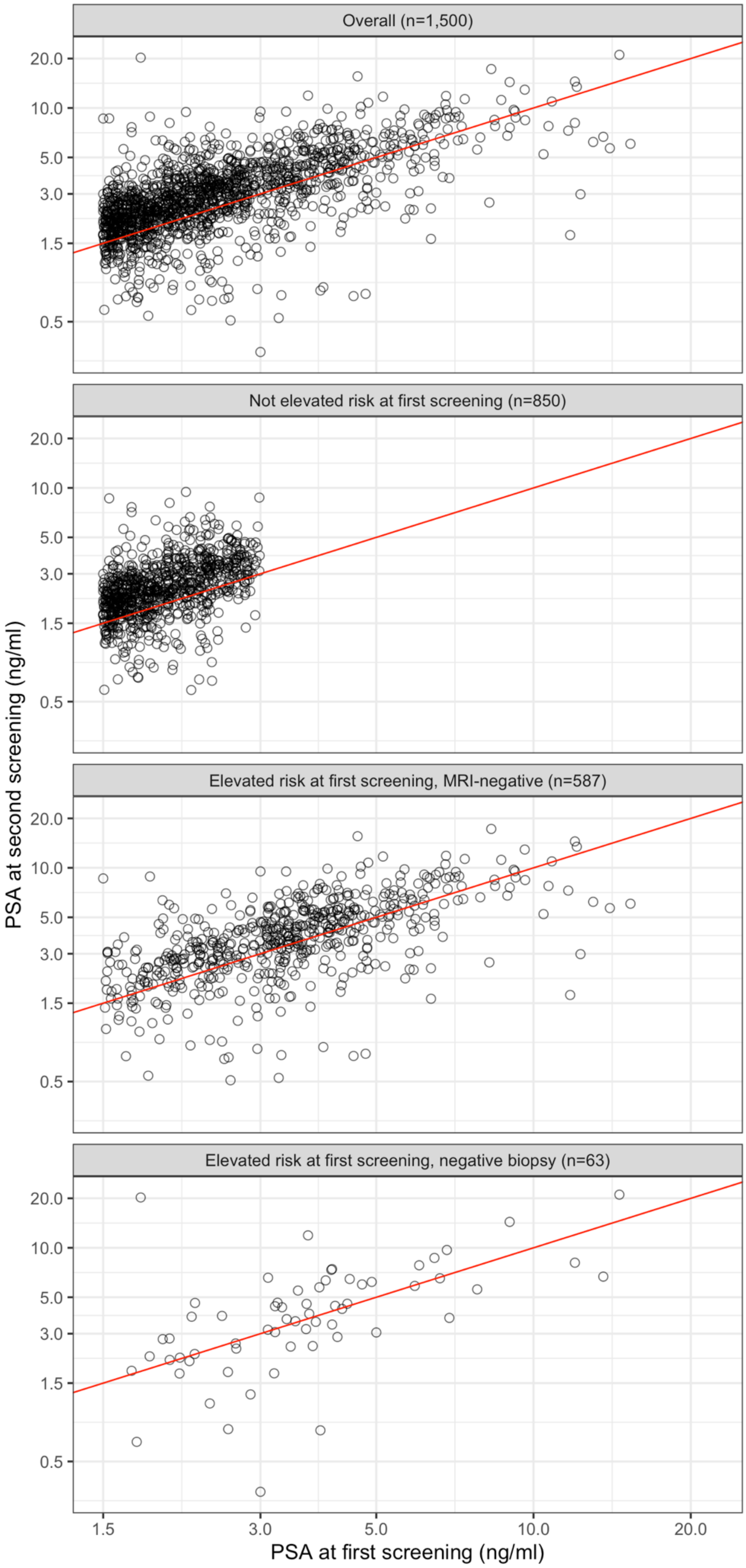

n=11 data points were excluded because of test failure.
Red lines are identity lines.

© 2024 Nordström T et al. *JAMA Network Open.*

eFigure 3. Number of Participants, Elevated Prostate-Specific Antigen (PSA) Test Results, Magnetic Resonance Imaging (MRI) and Biopsy Procedures Performed, and Detected Cancers Normalized to a Population of 10 000 Men Invited to a Second Screening

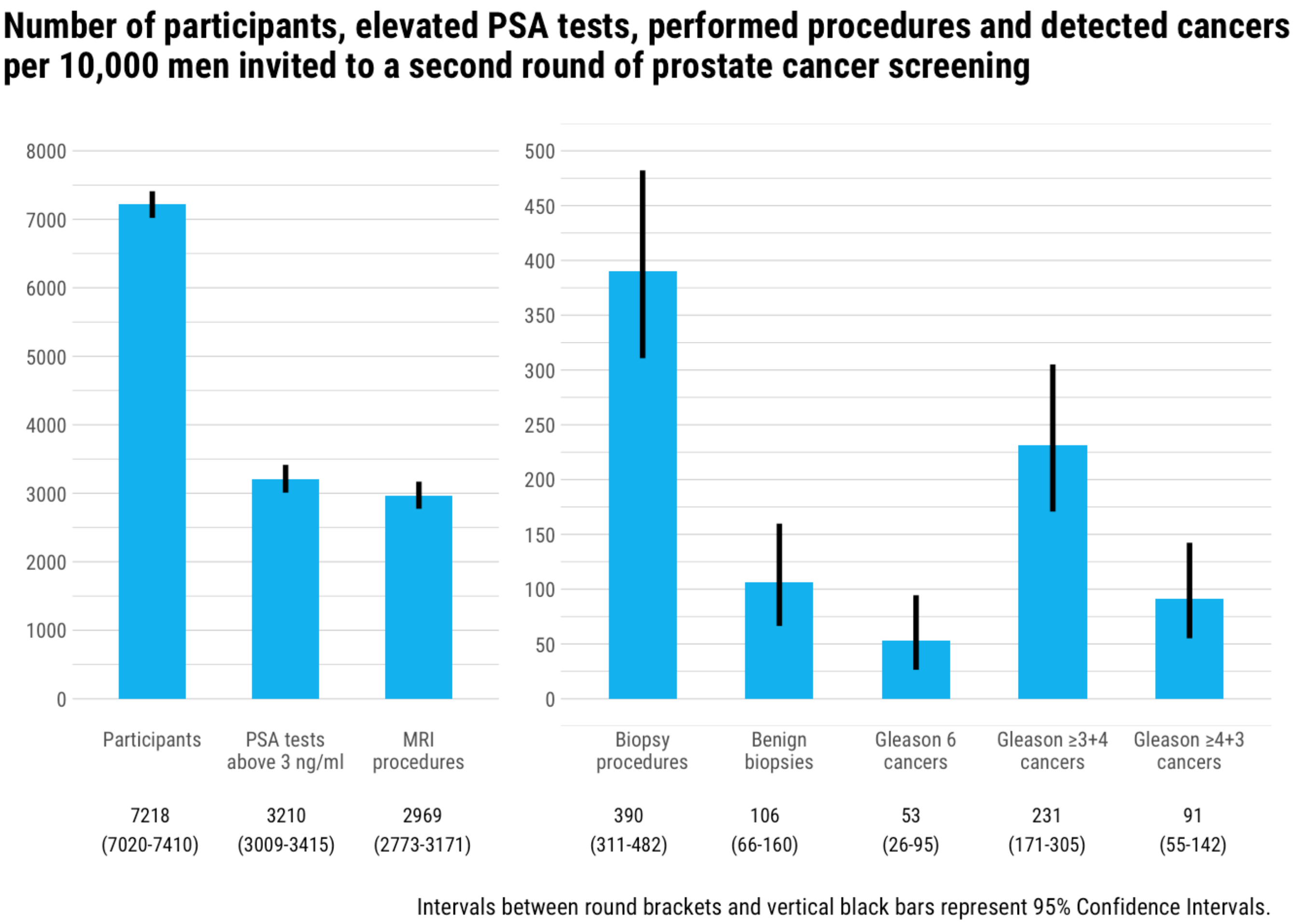

© 2024 Nordström T et al. *JAMA Network Open.*
