# Supplementary material for: Repeated Prostate Cancer Screening Using Prostate-Specific Antigen Testing and Magnetic Resonance Imaging: A Secondary Analysis of the STHLM3-MRI Randomized Clinical Trial
Source: JAMA Netw Open. 2024 Feb 7;7(2):e2354577. doi: 10.1001/jamanetworkopen.2023.54577 (PMC10851096; doi:10.1001/jamanetworkopen.2023.54577)
Supplement: Supplement 3. — Data Sharing Statement [file jamanetwopen-e2354577-s003.pdf]

Data Sharing Statement

Nordström. Repeated Prostate Cancer Screening Using Prostate-Specific Antigen Testing and Magnetic Resonance Imaging. *JAMA Netw Open*. Published February 07, 2024.
doi:10.1001/jamanetworkopen.2023.54577

Data
Data available: Yes
Data types: Deidentified participant data
How to access data: Tobias.nordstrom@ki.se
When available: beginning date: 01-01-2024, end date: 12-31-2025

Supporting Documents
Document types: Statistical/analytic code
How to access documents: Tobias.nordstrom@ki.se
When available: With publication

Additional Information
Who can access the data: researchers whose proposed use of the data has been approved
Types of analyses: For validation of study results
Mechanisms of data availability: After approval of proposal and with signed data share agreement
